# Genetic variability in landraces populations and the risk to lose genetic variation. The example of landrace ‘Kyperounda’ and its implications for *ex situ* conservation

**DOI:** 10.1371/journal.pone.0224255

**Published:** 2019-10-29

**Authors:** Angelos C. Kyratzis, Nikolaos Nikoloudakis, Andreas Katsiotis

**Affiliations:** 1 Department of Vegetable Crops, Agricultural Research Institute, Nicosia, Cyprus; 2 Department of Agricultural Science, Biotechnology and Food Science, Cyprus University of Technology, Limassol, Cyprus; Aristotle University of Thessaloniki, GREECE

## Abstract

Genetic characterization enhances the development of rational conservation strategies and the utilization of germplasm to plant breeding programs. In the present study, 19 microsatellite markers were employed to evaluate the genetic diversity and the genetic affiliations across 20 Cypriot durum wheat (*Triticum turgidum L*. subsp. *durum*) landraces, 13 landraces from the broader Mediterranean basin and 22 modern varieties. Cluster analysis depicted a clear separation among modern varieties and landraces, regardless of their origin. Landraces presented the highest genetic variation (average discriminating power of 0.89) and a high number of private alleles (131) was detected; underlying the unique genetic mark-up of this genepool. AMOVA revealed that the highest variability was detected within the landraces originating from Cyprus and landraces from the broader Mediterranean basin. The Cypriot landrace ‘Kyperounda’ was selected for further evaluation of its’ intra-genetic variation and it was determined that genetic diversity was higher in accessions conserved as sublines (He 0.643–0.731) than bulks (He 0.384–0.469). Bayesian analysis revealed substantial admixture within ‘Kyperounda’ accessions, depicted also by Principal Coordinate Analysis. The findings of the current manuscript emphasize that high intra-genetic diversity is retained when landraces are conserved as sublines in *ex situ* collections, while landraces that are conserved as bulks have a higher risk of bottleneck. Hence, a more exhausting diversity evaluation is needed in order to fully utilize landraces in breeding schemes and to prevent the loss of genetic variation.

## Introduction

The center of wheat domestication is located at the Fertile Crescent, where its cultivation is evident since 12.000 Before Present [1], gradually spreading to adjacent areas, including Cyprus [2]. Among the various tetraploid forms, durum wheat (*Triticum turgidum* L. subsp. *durum*) developed a definite agricultural significance and gradually predominated its ancestors [1]. The long history of durum wheat in the area, in combination with the diverse edaphic and climatic conditions, resulted to the formation of highly diverse landraces [3–6]. In the case of Cyprus, a significant number of durum wheat landraces, having a high phenotypic variation, were cultivated until the 70s. Among these, stood out the landrace ‘Kyperounda’ that was appreciated because of its adaptation to the local environment, distinctive quality traits and a high phenotypic variability [7,8].

Despite their good adaptation to local conditions, resulting from their long presence in the area [9], landraces were gradually replaced by the more productive modern varieties, especially under high yielding environments [4,6,10]. Fortunately, before becoming obsolesce, landraces were collected and are now mainly *ex situ* conserved in national, regional, and international genebanks. Historically, two main strategies have been followed for the collection of wheat landraces: either creating bulks from random sampling of individual spikes or collecting individual spikes based on phenotypic differences (ear lines or sublines) [11].

Nowadays, there is an increasing trend of reviving the forgotten landraces via their utilization in plant breeding programs. Given their millennia evolutionary history and adaptation to low-input agricultural systems, landraces can indeed contribute to breed novel varieties with improved productivity, adaptation, resilience to climate change, nutritional value [12,13] and quality attributes [14]. It is generally reported that landraces depict higher genetic variability than modern varieties [6,15], even though there are studies reporting otherwise [16,17]. Nevertheless, all studies converge to the conclusion that landraces have discrete genetic compositions from modern varieties [18–21]. Thus, landraces constitute a genepool of unexplored alleles [22], and their introgression to breeding programs could contribute to the broadening of the genetic base and to the improvement of ‘next generation’ wheat varieties [23].

Genetic characterization with robust molecular markers, such as microsatellites [24–29], allows the development of rational strategies for *ex situ* conservation and enhances the germplasm use in breeding programs [5,30–32]. Data from molecular markers provide the basis for the construction of core collections [23,33] and the designation of genetically unique accessions. This further facilitates the integration of regional and global initiatives, such as, the European Genebank Integrated System (AEGIS). Most importantly, the acquired levels of genetic proximity between crossing genotypes provides a baseline projection regarding attributes of the expected offsprings [19,31]. Despite the fact that several studies were conducted over the last years aiming at the genetic characterization of durum wheat genetic resources [5, 6,19], a large proportion of *in situ* and *ex situ* genetic variation in landraces collections, remains undiscovered [23,30,32]. In the case of Cyprus, a very limited number of durum wheat landraces originating from the island were included in previous studies aiming to investigate the genetic diversity and structure of durum wheat genetic resources [4, 6, 22, 31]. As a result, there is no coherent information regarding the genetic constituents within Cypriot durum wheat landraces. Nonetheless, genotyping a large number of accessions per country is still critical, as it contributes to understand the evolutionary relationships of wheat landraces [30].

Although by definition landraces are genetically diverse dynamic populations [9], the knowledge about their intra-variability is limited [34]. Indeed, the majority of studies so far aimed to evaluate the genetic diversity and the genetic relations between accessions, thus sampling was not exhausting and only referred to a few individuals per landrace. However, it is well established that separate analysis of several individuals is needed in order to accurately determine the within genetic diversity of landrace accessions [35]. In the case of durum wheat, a limited number of studies were aimed to investigate the within genetic diversity of discrete landraces [14,33,36–39], or how intra genetic diversity can be affected by the *ex situ* conservation method [30]. Still, such information is crucial for preventing the loss of genetic variation in *ex situ* conservation and superficial estimations could undermine the full exploitation of landraces in plant breeding schemes.

The current study is divided in two sections. The first part aims to evaluate the genetic diversity across Cypriot durum wheat landraces (*Triticum turgidum L*. subsp. *durum*), landraces from the broader Mediterranean basin and modern varieties. Furthermore, we aim to in-depth describe their genetic affiliations, as a first step to enhance their use to plant breeding programs in the Mediterranean Basin. The second part focuses on the Cypriot landrace ‘Kyperounda’, which was the predominant landrace in Cyprus, known for its high phenotypic diversity and adaption to hot and dry winds and a very wide variety of soil types [7,8]. This landrace was selected for further investigation of its genetic variation (via higher density screening of genotypes) across accessions collected at different time periods, environments and regeneration methods. The implications of *ex situ* conservation and the within genetic diversity of durum wheat landrace populations are discussed.

## Materials and methods

### Plant material

#### Genetic diversity and genetic affiliations across accessions

Fifty-five durum wheat (*Triticum turgidum* subsp. *durum*) accessions were selected for the present study, the majority being landraces originated from Cyprus and other Mediterranean countries. These accessions were assigned into four groups, according to distinct origin, category and conservation method (Table 1). Group I was composed of six varieties developed by the national breeding program in Cyprus, representing the main commercial varieties cultivated in the island for the last 40 years. Group II contained 16 varieties widely cultivated in the Mediterranean Basin (developed by non-Cypriot breeding programs). Group III, the largest group, included 20 Cypriot landraces, most of them conserved at the genebank of the Agricultural Research Institute (ARI), representing the known durum wheat landraces of Cyprus. Finally, Group IV contained 13 landraces originating from other Mediterranean countries, kindly provided by the International Center for Agricultural Research in the Dry Areas (ICARDA) genebank. The variety ‘Chinese spring’ (*Triticum aestivum* L.), provided by the Leibniz-Institut für Pflanzengenetik und Kulturpflanzenforschung (IPK) genebank was also included as an outgroup.

**Table 1:** List of the accessions used in the present study, conservation method and average number of alleles per primer and accession.

Number	Origin / Registration Country	Accession name / identity	Category	Group	Conservation method	Average no. of alleles per primer and accession
1.	Cyprus	Aronas	VAR	I	-	1.21
2.	Cyprus	Mesaoria	VAR	I	-	1.32
3.	Cyprus	Karpasia	VAR	I	-	1.21
4.	Cyprus	Makedonia	VAR	I	-	1.21
5.	Cyprus	Ourania	VAR	I	-	1.21
6.	Cyprus	Hekabe	VAR	I	-	1.16
7.	ICARDA	Ammor 6	VAR	II	-	1.26
8.	Syria	IG129081	VAR	II	-	1.37
9.	Greece	Anna	VAR	II	-	1.21
10.	Greece	Atlas	VAR	II	-	1.21
11.	Greece	Matt	VAR	II	-	1.32
12.	Greece	Mexikali 81	VAR	II	-	1.21
13.	Greece	Pisti	VAR	II	-	1.21
14.	Italy	Simeto	VAR	II	-	1.37
15.	Italy	Duilio	VAR	II	-	1.16
16.	Italy	Iride	VAR	II	-	1.16
17.	Italy	Claudio	VAR	II	-	1.21
18.	Italy	Svevo	VAR	II	-	1.26
19.	ICARDA	Adnan2	VAR	II	-	1.21
20.	ICARDA	Omrabi5	VAR	II	-	1.42
21.	ICARDA	Korifla	VAR	II	-	1.37
22.	ICARDA	Waha	VAR	II	-	1.16
23.	Cyprus	Kyperounda ARI00002	LR	III	Sublines	2.79
24.	Cyprus	Kyperounda ARI00030	LR	III	Sublines	2.79
25.	Cyprus	Kyperounda ARI00062	LR	III	Sublines	2.32
26.	Cyprus	Kyperounda Br*	LR	III	Pure line	1.32
27.	Cyprus	Maurotheri ARI00020	LR	III	Sublines	2.63
28.	Cyprus	Maurotheri ARI00061	LR	III	Sublines	3.00
29.	Cyprus	Maurokyperounda ARI00099	LR	III	Sublines	1.58
30.	Cyprus	IG96271	LR	III	Bulk	2.63
31.	Cyprus	Psathas ARI00007	LR	III	Sublines	2.00
32.	Cyprus	Tripolitiko ARI00024	LR	III	Sublines	2.26
33.	Cyprus	Famira ARI00027	LR	III	Sublines	1.95
34.	Cyprus	Famira ARI00076	LR	III	Sublines	3.05
35.	Cyprus	Famira Far**	LR	III	Farmers	2.05
36.	Cyprus	Loizos ARI00084	LR	III	Sublines	2.05
37.	Cyprus	Irakinos ARI00106	LR	III	Sublines	1.79
38.	Cyprus	Kokkino ARI00095	LR	III	Sublines	2.32
39.	Cyprus	Kampouriko ARI00102	LR	III	Sublines	2.16
40.	Cyprus	Aspris ARI00104	LR	III	Sublines	1.74
41.	Cyprus	Broulias ARI00017	LR	III	Sublines	3.47
42.	Cyprus	IG127457	LR	III	Bulk	1.63
43.	Spain	IG84979	LR	IV	Bulk	1.74
44.	Azerbaijan	IG140526	LR	IV	Bulk	1.63
45.	Greece	IG85710	LR	IV	Bulk	1.63
46.	Iran	IG86179	LR	IV	Bulk	1.42
47.	Israel	IG86653	LR	IV	Bulk	2.00
48.	Syria	IG95789	LR	IV	Bulk	1.84
49.	Algeria	IG97359	LR	IV	Bulk	1.63
50.	Livia	IG98726	LR	IV	Bulk	1.32
51.	Israel	IG83901	LR	IV	Bulk	1.21
52.	Tunisia	IG99151	LR	IV	Bulk	1.37
53.	Armenia	IG126364	LR	IV	Bulk	1.58
54.	Jordan	IG97193	LR	IV	Bulk	1.58
55.	Morocco	IG96437	LR	IV	Bulk	1.32
56.	IPK	Chinese spring***			-	1.37

LR = Landraces. VAR = Varieties,

* Conserved by national breeding program,

** Collected from farmers in 2011,

***Triticum aestivum

Landrace accessions in genebanks can be conserved as bulk (seeds from different ears are mixed together) or sublines (each ear is conserved separately). The landraces at the ARI genebank were collected during 1978 and are conserved as sublines i.e. 50–150 distinct ear lines, for each accession, depending on the phenotypic variability observed at the collecting site [40]. The landraces in ICARDA genebank are conserved as bulks. When conserved as sublines, 10 seeds were randomly selected from each subline in order to create an accession bulk, and 100 seeds were drawn from the accession bulk for sowing. When conserved as bulk, 100 seeds were randomly selected from each accession for sowing. All accessions were cultivated to single plots in the same field at Athalassa experimental station (35°08´N, 33°24´E). Weeds were controlled and additional irrigation was supplied to avoid water stress. Off-types and hexaploid plants were discarded from the plots. After harvesting, 60 seeds from each plot (accession) were randomly selected and grown in a glasshouse. DNA was extracted from a bulk containing tissue from around 50 seedlings per accession.

#### Intra-genetic diversity and genetic affinity of ‘Kyperounda’ accessions

Five ‘Kyperounda’ accessions were selected to further investigate the intra-genetic diversity and their genetic affinity. Three accessions were selected from the germplasm conserved at the ARI genebank (representing three distinct regions with diverse environmental conditions) and two accessions from entries conserved and kindly provided by USDA-ARS (National Small Grains Collection) (Table 2). The latter were selected because they were collected at least 20 years prior to the collection of the genetic material conserved at the ARI genebank.

**Table 2:** List of accessions (populations) of the landrace ‘Kyperounda’, conservation method and number of sublines / plants used for phenotyping / genotyping (2i); meteorological data from the collecting sites of three accession (populations) conserved at ARI genebank (2ii).

**Table 2(i)**	**Accession No.**	**Collecting site**	**Registration**	**Conservation method**	**No of sublines / plants phenotyped**	**No of sublines / plants genotyped**
Population 1	ARI00002	Athienou	1978	Sublines	51*	40*
Population 2	ARI00030	Neo Chorio Pafou	1978	Sublines	54*	52*
Population 3	ARI00062	Pareklisia	1978	Sublines	55*	53*
Population 4	PI210951	unknown	1953	Bulk	46**	46**
Population 5	PI210960	unknown	1953	Bulk	26**	26**
**Table2(ii)**	**Average maximum temper (°C) (Nov–Feb)**	**Average maximum temper (°C) (Mar–May)**	**Average minimum temper (°C) (Nov–Feb)**	**Average minimum temper (°C) (Mar–May)**	**Average precip (mm) (Oct–Feb)**	**Average precip (mm) (Mar–May)**
Population 1	17.63	24.30	7.10	10.67	229.10	74.40
Population 2	18.19	21.95	9.25	11.40	350.10	76.80
Population 3	20.25	24.97	8.43	11.57	351.80	79.00

*Number of sublines,

**Number of plants

The accessions in ARI genebank are conserved as sublines. Forty seeds were sown from each subline to one-meter-long rows at the Athalassa experimental station. In total, 160 sublines from the three ARI accessions were examined (Table 2). Off-types and hexaploids were removed. For each row, heading dates and plant heights were recorded, ears were hand-harvested and evaluated for distinctive morphological characteristics; i.e. length of the beak, ear color and glume hairiness. DNA was extracted from one seedling per subline. The two accessions from USDA are conserved as bulks. Seeds from these accessions were sown in single plots at Athalassa experimental station to ensure that off-types present in the accessions were removed. One leaf from each plant was collected for DNA extraction (Table 2).

### DNA extraction and PCR amplification

#### Genetic diversity and genetic affiliations across accessions

Genomic DNA was extracted using the Invisorb^®^ Spin Plant Mini Kit (STRATEC Biomedical AG, Birkenfeld, Germany), following the manufacturer’s instructions. DNA concentration and quality was determined by Nanodrop 1000 (Thermo Scientific, Wilmington, USA) and verified in agarose electrophoresis. Nineteen microsatellite markers (SSRs) were selected based on their polymorphism and chromosomal location. These markers have been previously described [24–27] (S1 Table).

It has been established that durum wheat fields grown with landraces are frequently contaminated with hexaploid wheats [7,33,41,42]. In the present study, alongside to the removal of hexaploid plants from the single plots based on phenotypic observations, primers WMS52 and WMC233 were also employed in order to verify the absence of hexaploid admixtures across genotypes. These primers are exclusively located in the D genome [25,26]. For PCR conducted, the hexaploid varieties ‘Gavdos’ and ‘Chinese Spring’ were also included as positive controls.

Amplification reactions were set up in a 25 μl volume of a mixture containing 50 ng of genomic DNA, 1x Type-it^®^ Multiplex PCR master mix (Type-it^®^ Microsatellite PCR kit, Qiagen, Venlo, Netherlands) and 0.2 μM of each primer (the forward primers were 5´-end labeled with FAM—5-carboxy-fluorescent). PCR amplification was performed in a PTC-200 thermocycler (Bio-Rad, Hercules, USA) under the following temperature profile: 5 min at 95°C, followed by 30 cycles (40 cycles were used for primer WMC161), each one including 30 sec at 95°C, 90 sec at annealing temperature depending of the primer pair (S1 Table), 30 sec at 72°C and a final extension step for 30 min at 60°C. A negative control was included in each set of PCR amplification. A subset of accessions (10%) was amplified twice to check the reproducibility of the primers.

Amplified PCR products were run on an ABI3130 genetic analyzer (Applied Biosystems, Foster City, CA, USA). Size standard GeneScan^™^ 500LIZ^®^ (Applied Biosystems) was added to each sample to delineate allele sizes. Data were analyzed using GeneMapper Software version 4.1 (Applied Biosystems, Foster City, CA, USA).

#### Intra-genetic diversity and genetic affinity of ‘Kyperounda’ accessions

The DNA extraction procedure was as described above. Five primer pairs (BARC 74, WMC 104, WMS 268, WMS 5 and WMC 89) were selected from the complete set of the 19 microsatellite markers, based on the number of alleles detected in the ‘Kyperounda’ and ‘Maurotheri’ accessions (Table 3). Amplification reactions were set up in a 10 μl volume of a mixture containing 25 ng of genomic DNA, 0.5 U Kapa Taq (Kapa Biosystems, Wilmington, MA, USA), 1x Kapa buffer A, 0.2 μM of each primer (the forward primers were 5´-end labeled with FAM—5-carboxy-fluorescent) and 0.2 mM dNTPs. PCR amplification was performed in a PTC-200 thermocycler (Bio-Rad, Hercules, USA) under the following temperature profile: 5 min at 95°C, followed by 35 cycles, each one included 30 sec at 95°C, 30 sec at 57°C, 30 sec at 72°C and a final extension step for 15 min at 60°C. Analysis of the amplification products was conducted as previously described.

**Table 3:** Number of alleles per primer and accession of ‘Kyperounda’ and ‘Maurotheri’ entries, total number of alleles and number of different alleles per primer in all accessions.

Accession name / identity	Kyperounda ARI00002	Kyperounda ARI00030	Kyperounda ARI00062	Maurotheri ARI00020	Maurotheri ARI00061	Mauro kyperounda ARI00099	Kyperounda Br*	Total	No. of different alleles
WMS268	7	7	4	5	9	2	2	36	12
WMC104	4	5	3	5	5	2	2	26	5
WMS5	5	4	2	4	5	1	1	22	6
BARC74	3	4	3	4	5	1	1	21	7
WMC89	4	2	3	3	3	3	2	20	5
WMS312	3	3	3	3	3	2	2	19	4
WMS148	3	3	4	2	3	2	1	18	4
WMS299	2	3	3	3	4	1	1	17	5
WMS619	2	3	3	4	2	2	1	17	5
WMS752	2	3	3	2	3	2	2	17	3
WMS46	2	3	2	3	3	2	1	16	4
WMS304	3	2	2	2	2	2	2	15	3
WMS169	2	2	2	2	2	1	1	12	3
WMS155	2	2	1	2	2	1	1	11	3
WMC161	3	2	1	1	1	1	1	10	3
WMC83	2	2	1	1	1	1	1	9	2
WMS260	1	1	1	1	2	2	1	9	2
WMS540	1	1	2	2	1	1	1	9	2
WMS389	2	1	1	1	1	1	1	8	2

* Conserved by the national breeding program as breeding line

### Data analysis

Multi-alleles were detected on the landraces bulked accessions composed of several plants. Hence, for the analysis of bulked accessions we applied a model (conversion of all allele fragments to a binary matrix) that does not require evolutionary assumptions in order to calculate genetic affiliations among accessions. On the other hand, analysis in Kyperounda populations was conducted on allele fragments (DNA was extracted from single plants and maximum two alleles per locus were detected).

#### Genetic diversity and genetic affiliations across accessions

As DNA extraction was performed on a bulk sample containing tissue from around 50 seedlings per accession, the average number of alleles per primer and accession was calculated as an indicator of the heterogeneity within accessions. The discriminating capacity and the level of polymorphism /informativeness described by the indexes of number of alleles, number of rare alleles, allele range, number of private alleles, Discriminating Power (Dj), and Resolving Power (RP) were calculated as previously reported [43–44]. The Dj represents the probability that two randomly chosen accessions are distinguishable from each other and it is considered an extension of Polymorphic Information Content (PIC), while RP represents the ability of a primer to distinguish between accessions. Analysis of Molecular Variance (AMOVA) was also performed to assess the within and between variance across groups using GenAlEx 6.4 [45]. The significance of the resulting variance components and the inter-population genetic distances were tested using 999 random permutations. In order to depict genetic associations, a Maximum Likelihood analysis (with supported bootstrapped values above 50%) was conducted using the SH-aLRT algorithm implemented in the IQ-TREE (ver. 1.6.11) software [46].

#### Intra-genetic diversity and genetic affinity of ‘Kyperounda’ accessions

The five ‘Kyperounda’ accessions were treated as populations. An analysis of variance (ANOVA) for heading date was carried between the three ARI populations conserved as sublines. Mean comparison was done with the Tuckey-b test. Box plots were constructed to depict the variation for heading data and plant height. Box plots and ANOVA were performed with SPSS V.22 (IBM).

Principal Coordinate Analysis (PCoA) was conducted to determine the associations between sublines / plants of the five populations. AMOVA was carried out to assess the within and between variance across populations. AMOVA, PCoA and the calculation of genetic variation indices (number of different alleles, number of effective alleles, rare alleles, number of unique alleles, expected heterozygosity, fixation indices and inbreeding coefficients) were performed using GenAlEx 6.4 [45]. The Bottleneck software was employed to identify possible bottlenecks using the Two Phase Model (TPM) and the Step-wise Mutation Model (SMM) [47], which are the most appropriate when using microsatellite loci [48], and the Wilcoxon sign-rank test is presented. The Putative population structure was analyzed using Structure 2.3.4 [49]. The structure software was run using the admixture model, with 10 independent replicate runs per K value (number of clusters) ranging from 1 to 10. Each run involved a burning period of 100,000 iterations and a post burning simulation length of 100,000. Validation of the most likely number of clusters K was performed with the Structure Harvester (https://taylor0.biology.ucla.edu/structureHarvester). A subline / plant was considered to belong to a cluster if its membership coefficient was ≥ 0.8 [6,31].

## Results

### Genetic diversity and genetic affiliations across accessions

**Fig 1:**
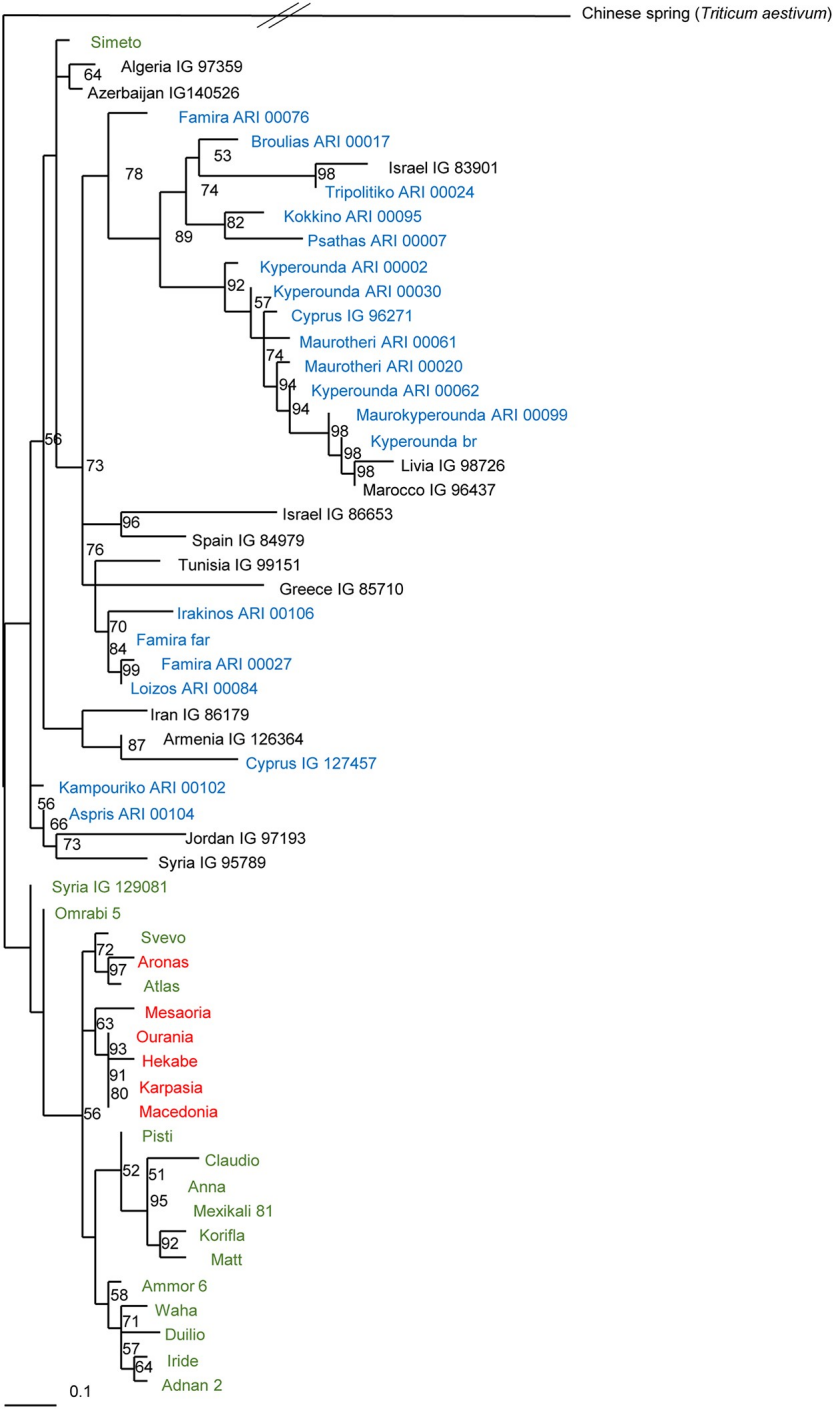
Bootstrapped dendrogram of 56 durum wheat accessions based on Maximum Likelihood analysis using the SH-aLRT algorithm. With red color: varieties bred by the national breeding program of Cyprus; with green color: varieties bred by other breeding programs in the Mediterranean; with blue color: landraces originated from Cyprus; and with black color: landraces originated from other areas in the Mediterranean Basin.

Modern varieties were found genetically homogeneous in comparison to landraces. The average number of alleles per primer and accession was 1.99 and 1.25 for landraces and modern varieties, respectively. The landraces conserved as sublines exhibited higher heterogeneity compared to the landraces conserved as bulks (Table 1); their average number of alleles per primer and accession was 2.28 and 1.56, respectively. Particularly high heterogeneity was also observed within the ‘Kyperounda’ and ‘Maurotheri’ accessions; these are phenotypically and genetically close (Fig 1). Altogether, 80 different alleles were detected in the seven accessions (Table 3), out of which, 16 alleles were exclusive to one accession. Primer WMS268, followed by primers BARC74 and WMS5, were highly polymorphic, detecting together 25 alleles. The lowest heterogeneity was detected within the ‘Kyperounda Br’ accession that is conserved by the national breeding program of Cyprus as a pure line. From the accessions provided by ICARDA, the highest heterogeneity was observed within accession ‘IG96271’. This accession was found to be genetically close to ‘Kyperounda’ (Fig 1).

All microsatellite primers used in the present study were found to be polymorphic (Table 4). The average discriminating power (Dj) was 0.84, 0.89, and 0.55 for the whole set of accessions, landraces, and modern varieties, respectively. The corresponding Resolving Power (RP) was 3.11, 3.61 and 1.67. Dj and RP for each primer were higher in landraces, except for primer WMS260. The WMS540 locus was found polymorphic in the group of landraces and monomorphic in the group of modern varieties. Three microsatellite loci (WMS752, WMS304 and WMC89) consistently produced at least two alleles per accession. These primers have been mapped to more than one locus (Grain genes database, https://wheat.pw.usda.gov/GG3/) (S1 Table).

**Table 4:** Levels of polymorphism detected by SSRs for the accessions studied.

	All accessions (56 accessions)					Landraces (33 accessions)					Modern Varieties (22 accessions)				
SSR	No of alleles	Nr	Range (bp)	Dj	RP	No of alleles	No of private alleles*	Range (bp)	Dj	RP	No of alleles	No of private alleles**	Range (bp)	Dj	RP
WMS752	14	4	105–161	0.93	4.43	13	7(2.2)	105–159	0.94	4.91	7	1(0.1)	105–161	0.73	2.64
WMS268	35	14	182–276	0.92	6.75	33	26(7.5)	182–276	0.99	9.27	9	2(1.0)	197–256	0.48	1.82
WMS312	15	8	184–246	0.82	3.18	14	13(6.4)	184–246	0.87	3.27	2	1	184–223	0.25	0.55
WMS148	9	2	139–167	0.89	2.89	8	4(0.2)	139–167	0.93	3.09	5	1	141–167	0.64	2.55
WMS619	12	5	135–164	0.88	2.93	10	9(1.3)	135–164	0.92	3.39	2	1	145–153	0.50	1.09
WMS5	8	-	158–176	0.93	3.54	7	3(0.1)	162–176	0.95	4.48	5	1(1.0)	158–170	0.80	2.00
WMS155	8	1	124–142	0.70	2.07	8	6(1.2)	124–142	0.89	2.85	2	0	124–128	0.09	0.18
WMS299	11	4	188–221	0.82	2.71	9	7(2.2)	188–221	0.89	3.21	3	1	192–215	0.64	1.91
WMS389	10	3	115–134	0.87	2.50	8	5(0.0)	115–134	0.92	2.85	4	1(1.0)	115–128	0.54	1.45
WMC161	16	9	137–185	0.80	3.25	14	9(4.4)	137–185	0.88	3.58	5	0	153–179	0.59	2.36
WMC89	9	3	124–178	0.89	3.86	6	2(1.0)	124–145	0.92	4.12	5	1	126–176	0.71	2.09
WMS304	9	1	196–216	0.87	2.93	8	2(0.1)	196–210	0.87	3.03	6	0	196–208	0.79	2.36
BARC74	13	6	146–187	0.88	3.04	10	6(0.5)	157–187	0.93	3.70	5	1(0.1)	167–177	0.64	1.82
WMS540	6	3	112–127	0.64	1.64	6	5(2.0)	112–127	0.76	2.26	1	0	114	0	0
WMS169	8	3	185–205	0.82	2.32	7	5(2.0)	185–197	0.85	2.55	3	1(1.0)	189–205	0.56	1.82
WMC104	14	5	120–188	0.90	4.11	14	10(3.1)	120–188	0.95	4.55	4	0	146–184	0.62	2.73
WMC83	8	2	95–167	0.71	1.75	7	5(0.2)	95–163	0.82	2.24	2	0	95–163	0.37	0.91
WMS260	6	2	143–166	0.78	2.25	4	1(1.0)	143–149	0.63	1.39	4	1(1.0)	145–166	0.71	2.00
WMS46	13	7	157–187	0.89	3.00	12	6(2.2)	157–185	0.95	3.94	6	0	171–183	0.59	1.36
Mean	11.79	4.56		0.84	3.11	10.42	6.89		0.89	3.61	4.21	0.68		0.55	1.67
Total	224	49				198	131				80	13			

Nr = Number of alleles with a frequency ≤5%, Dj = Discriminating power, Rp = Resolving power.

*first number in brackets: private alleles in landraces originating from other areas; second number in brackets: private alleles in landraces originating from Cyprus.

** first number: private alleles in varieties originating from other breeding programs; second number: private alleles in varieties originating from Cypriot breeding program.

In total, 224 alleles were detected with an average of 11.79 alleles per locus. Forty-nine alleles were classified as rare, since they appeared with a frequency lower than 0.05. Landraces had a higher number of private alleles compared to modern varieties (Table 4). Thirty-six and 34 private alleles were detected in landraces originating from Cyprus and elsewhere, respectively.

Analysis of molecular variance (AMOVA) revealed that 77% of the total variation was attributed to the genetic variation among accessions within groups, 16% to the genetic variation among landraces and modern varieties, while the remaining 7% to the genetic variation among groups within landraces and modern varieties (Table 5). The three sources of variation were significant (*PhiPT = 0*.*230*, *PhiRT = 0*.*165*, *PhiPR = 0*.*078* respectively; *p = 0*.*001*). The highest variability was recorded for landraces originating from Cyprus (Group III; 50.27%), followed by landraces from other Mediterranean countries (Group IV; 27.54%), modern varieties from other breeding programs (Group II; 17.78%) and modern varieties from Cyprus (Group I; 4.41%). The most diverged groups were varieties originating from foreign breeding programs, and Cypriot landraces (*PhiPT = 0*.*266; p = 0*.*001*), while genetic affinity was observed between the two landrace groups (*PhiPT = 0*.*052; p = 0*.*006*).

**Table 5:** Analysis of molecular variance. Pairwise comparisons between groups (*PhiPT* values) are shown.

Source	df	SS	MS	Estimated variance	Variance (%)
Among landraces /modern varieties	1	151.1	151.5	4.098	16%
Within landraces /modern varieties	2	78.12	39.06	1.624	7%
Within groups	51	978.4	19.18	18.184	77%
	Group 1	Group 2		Group 3	Group 4
Group 1		0.001*		0.001*	0.001*
Group 2	0.186			0.001*	0.001*
Group 3	0.254	0.266			0.006*
Group 4	0.188	0.157		0.052	

Lower diagonal = *PhiPT* Values,

*upper diagonal = p values computed with 999 permutations

Fig 1 depicts the bootstrapped dendrogram based on maximum likelihood analysis using the SH-aLRT algorithm. ‘Chinese Spring’, the only *Triticum aestivum* accession, was out-grouped from the core of durum wheat entries. With the exception of ‘Simeto’, all other modern varieties were clustered together. Landraces did not follow a specific geographical pattern. However, ‘Kyperounda’ and ‘Maurotheri’ accessions were clustered together alongside to ‘Cyprus-IG96271’, ‘LIVIA-IG98726’ and ‘MAROCCO-IG96437’.

### Intra-genetic diversity and genetic affinity of ‘Kyperounda’ accessions

**Fig 2:**
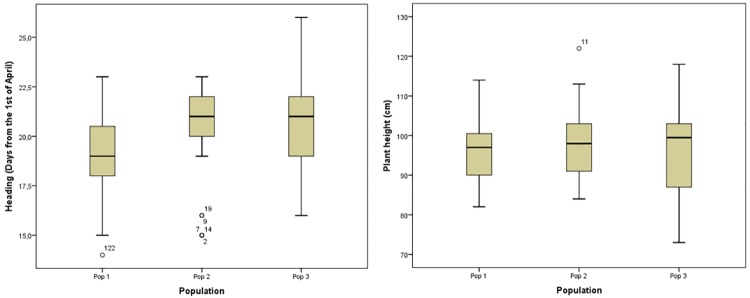
Variation between and within ‘Kyperounda’ populations conserved as sublines for heading date and plant height.

From the 160 ‘Kyperounda’ sublines conserved at ARI genebank and examined in the field plots, 15 sublines were discarded from further analysis as off-types or hexaploid wheat (Table 2i). Variation of heading date and plant height was detected (Fig 2). Specifically, ANOVA showed that that there were statistically significant differences between population (*p<0*,*0001*) in heading date; population 1 differed significantly from the other two populations. As expected, early heading sublines reached maturity earlier than late heading sublines (S1 Fig). Most sublines had dense, short, intermediate black colored spikes, without hairiness on glumes and short beak. However, deviations from ‘true types’ (sublines with hairs on the glumes, long beak and slightly or intense ear color) were also observed (S2 Fig). This declination was observed in all populations, even though the variation within populations 2 and 3 was greater (S3 Fig).

**Fig 3:**
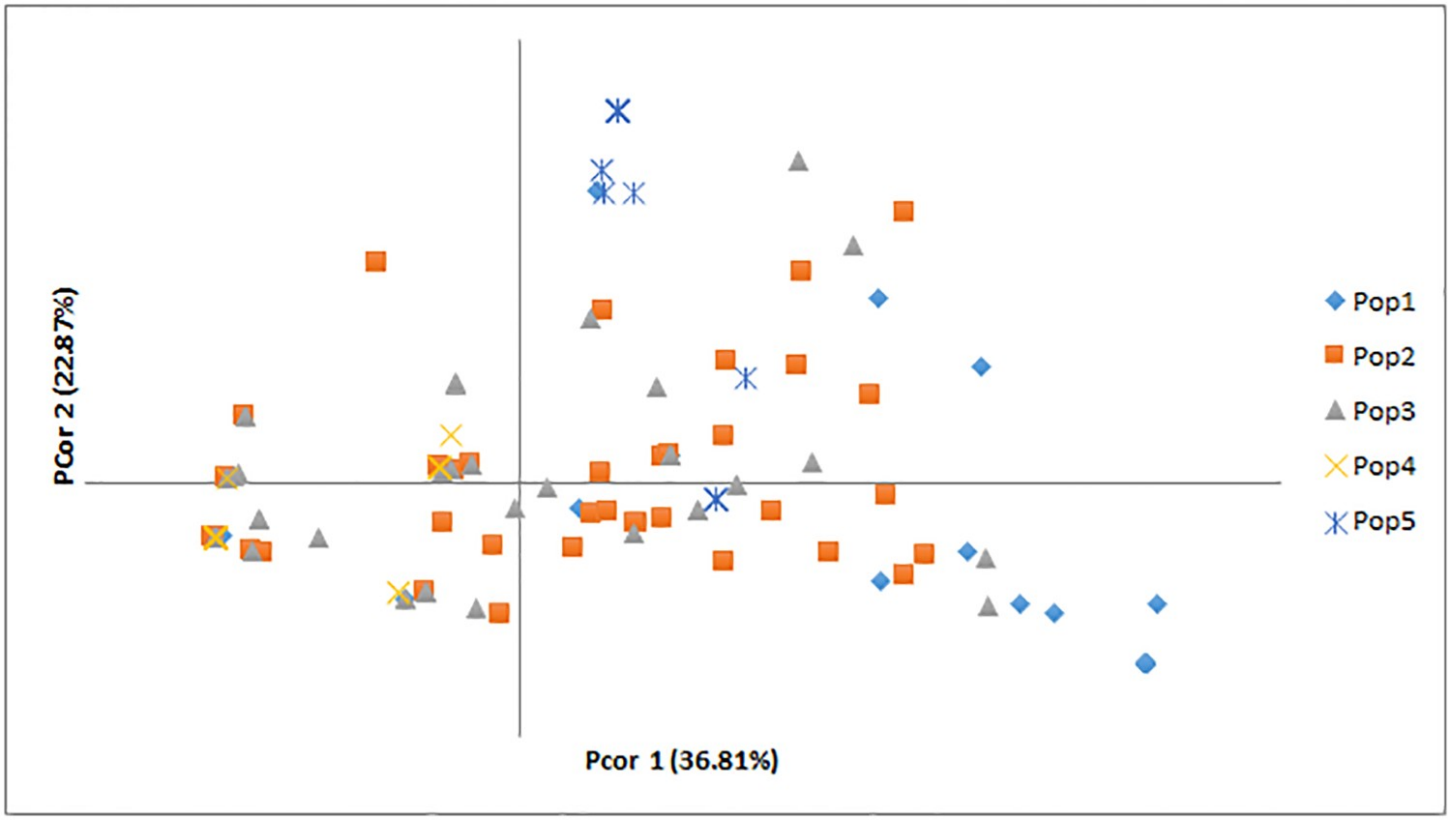
Principal coordinate analysis of ‘Kyperounda’ sublines / plants. Entries from different populations are shown with different color and marker.

A high level of polymorphism was revealed for all loci (S2 Table). Primer WMC89 consistently produced two alleles per subline/plant. Fifty-six alleles were recorded with an average of 11.2 alleles per locus. Thirty alleles, or 53.57% of the recorded alleles, were rare. In total, 50 discrete genotypes were detected, with 35 of them having a frequency less than 0.05. On the contrary, the most common genotype had a frequency of 0.281. Principal Coordinate Analysis revealed that 36.81% and 22.87% of the total diversity was explained by the first and the second axes, respectively (Fig 3). No definite grouping of the populations was observed.

AMOVA revealed that 75% of total variation was due to within population variation and the remaining 25% was attributed among populations (Table 6). The populations conserved as sublines (Populations 1, 2 and 3) depicted higher genetic variability than the populations conserved as bulks (Populations 4 and 5). Population 2 exhibited the highest variability (33.66%), followed by Population 3 (26.30%), Population 1 (24.38%), Population 5 (9.98%) and Population 4 (5.68%).

**Table 6:** Analysis of molecular variance. Pairwise comparisons between populations (*PhiPT* values) are shown.

Source	df	SS	MS	Estimated variance	Variance (%)
Among populations	4	198.84	49.71	1.08	25
Within populations	212	680.22	3.21	3.21	75
	Pop 1	Pop 2	Pop 3	Pop 4	Pop 5
Pop 1		0.001*	0.001*	0.001*	0.001*
Pop 2	0.147		0.054*	0.001*	0.001*
Pop 3	0.215	0.017		0.001*	0.001*
Pop 4	0.434	0.206	0.124		0.001*
Pop 5	0.381	0.308	0.371	0.621	

Lower diagonal = *PhiPT* Values,

*upper diagonal = p values computed with 999 permutations

The higher genetic variation within Populations 1, 2 and 3 is further evident by the genetic diversity of the polymorphism indices depicted in Table 7. The higher genetic diversity portrayed by Population 5 compared to Population 4, was also in line to field observations. Two distinct phenotypes (A: short beak and intensively coloured spike, B: long beak and intermediate coloured spike) were observed in Population 5, while Population 4 appeared uniform (short beak and intermediate coloured spike). With exception of Population 5, all other populations did not show evidence of recent bottleneck (TPM and SMM p values > 0.05) (S3 Table).

**Table 7:** Diversity indices in ‘Kyperounda’ populations.

	**Population 1**			**Population 2**			**Population 3**			**Population 4**			**Population 5**		
**Primer**	**Ne**	**He**	**F**	**Ne**	**He**	**F**	**Ne**	**He**	**F**	**Ne**	**He**	**F**	**Ne**	**He**	**F**
BARC 74	2.662	0.624	0.880	3.223	0.690	0.721	2.412	0.585	0.645	1.459	0.315	1.000	2.074	0.518	1.00
WMC104	2.805	0.643	0.456	3.997	0.750	0.051	3.025	0.669	-0.268	2.000	0.500	-1.000	2.000	0.500	0.846
WMS268	2.960	0.662	-0.472	4.422	0.774	-0.218	3.760	0.734	-0.311	2.089	0.521	-0.918	3.485	0.713	-0.402
WMS5	3.045	0.672	0.963	4.015	0.751	0.923	2.476	0.596	0.937	1.092	0.084	1.000	1.000	0	-
WMC89	3.159	0.683	-0.463	3.219	0.689	-0.451	2.710	0.631	-0.585	2.000	0.500	-1.000	2.585	0.613	-0.631
Mean	2.926	0.657	0.273	3.775	0.731	0.205	2.877	0.643	0.084	1.728	0.384	-0.184	2.229	0.469	0.203
SE	0.088	0.010	0.314	0.239	0.017	0.266	0.246	0.027	0.297	0.194	0.084	0.483	0.406	0.123	0.375
	**Population 1**			**Population 2**			**Population 3**			**Population 4**			**Population 5**		
**Primer**	**Na**	**Nr**	**Np**	**Na**	**Nr**	**Np**	**Na**	**Nr**	**Np**	**Na**	**Nr**	**Np**	**Na**	**Nr**	**Np**
BARC 74	7	3	1	9	4	0	9	4	1	2	0	0	3	1	0
WMC104	5	1	1	7	2	1	8	5	2	2	0	0	3	1	0
WMS268	9	6	2	12	8	4	10	6	2	4	2	0	4	0	0
WMS5	7	2	1	7	1	1	6	1	0	3	2	2	1	0	0
WMC89	7	4	1	6	2	0	6	3	0	2	0	0	3	0	0
Total	35	16	6	41	17	6	39	19	5	13	4	2	14	2	0

Ne = Number of effective alleles, He = Expected heterozygosity, F = Fixation Index, Na = Number of different alleles, Nr = Number of different alleles with a frequency ≤5%, Np = Number of alleles unique to a single population.

The least affinity among populations was found among Populations 4 and 5 (*PhiPT = 0*.*621; p = 0*.*001*), followed by Populations 1 and 4 (*PhiPT = 0*.*434; p = 0*.*001*), while the greatest genetic proximity was observed between Populations 2 and 3 (*PhiPT = 0*.*017; p = 0*.*054*) and Populations 3 and 4 (*PhiPT = 0*.*124; p = 0*.*001*) (Table 6).

**Fig 4:**
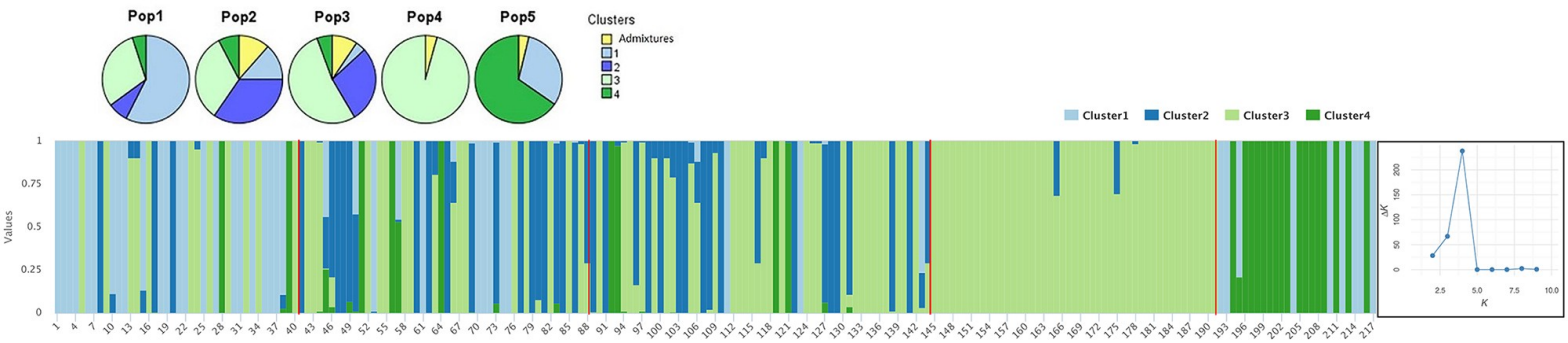
Population structure analysis of ‘Kyperounda’ populations with optimum cluster K = 4.

A Bayesian based approach was further used to examine the population structure and the allocation of the genetic diversity. The optimum for the *ad hoc* quantity, based on the second order rate of change of the likelihood function with respect to ΔK, was observed for K = 4 (Fig 4). Two hundred and three individuals out of 217 had a proportion of membership higher than 0.8. The analysis revealed substantial admixture within accessions; the most uniform population being Population 4, with almost all individuals belonging to cluster 3. The highest percentage of sublines from Population 3 were also grouped to cluster 3. Population 1 had the highest percentage of sublines grouped to cluster 1, while the highest percentage of individuals of Population 5 grouped to cluster 4. The majority of Population 2 sublines were affiliated to cluster 2 and 3. Populations 2 and 3 had the highest percentage of sublines with a membership proportion lower than 0.8 (admixtures).

## Discussion

### Genetic diversity and genetic affiliations across accessions

The presence of multiple products in microsatellite studies can be ascribed to within accession heterogeneity, segregation at the respective SSR marker [30] or to multilocus markers [50,51]. In the present study, the higher number of alleles per locus within landrace entries can be attributed to the genetic heterogeneity between individuals within an accession. Landraces are characterized by high heterogeneity, since the variability within populations serves as an important adaptive trait under stressful environments and low inputs [13,33]. Heterogeneity within landrace accessions is frequently reported in genetic studies [30,42,52,53,54], and in the present study heterogeneity was higher in landraces accessions conserved as sublines.

The 19 microsatellites employed were highly polymorphic revealing the existence of high genetic diversity. The variability calculated by means of descriptive statistical indexes, such as Dj and RP, was higher (almost two fold) in landraces when compared to modern varieties. This is the outcome of the larger heterogeneity in the lineage of landraces since they are mixtures of discrete genotypes. In particular, the mean Dj value was 0.89 for landraces and 0.55 for modern varieties (RP was 3.61 and 1.67 respectively; Table 4). Furthermore, in the case of landraces, Dj varied across loci ranging from 0.63 (WMS260) to 0.99 (WMS268), indicating a very low likelihood of alike genotypes. Hence, it seems that Dj is more appropriate than RP in order to select primers for genetic identification; since it relies on the number of assessed individual genotypes and allows the probability display of randomly-selected individuals’ discrimination by each marker [55].

The extent of variability revealed in this work is in agreement with previous studies in durum wheat landraces [4,5,52,56]. For example, Soriano et al. [6] used 44 SSRs and detected 448 alleles [with a mean of 10 alleles per locus (average expected heterozygosity: 0.71)], in a set of 192 accessions originating from the Mediterranean Basin. Correspondingly, in an analysis of 52 historic varieties and landraces of breed wheat, 263 alleles were identified with a mean of 10.5 alleles per locus (average PIC: 0.74), using 24 SSRs [53].

A moderate level of polymorphism was detected in modern varieties and it is comparable to previous studies for durum [18,28] and bread wheat modern varieties [50]. The higher levels of genetic diversity within landraces, in contrast to modern varieties, revealed in this and previous research [3,6,15,21,57], reinforce the potential use of landraces in order to widen the genetic base in modern varieties. Cypriot landraces had the highest genetic variability with 36 private alleles. The importance of islands, as isolators, to the accumulation of distinct genetic diversity and recessive forms was stressed in earlier studies [40,58]. For example, liguleless landraces, a rare trait in durum wheats, where recorded in Cyprus at the early years of the previous century [59].

Cluster analyses showed a clear differentiation between modern varieties and landraces [6,15,20,21], except from ‘Simeto’ that was bred from crosses involving landraces [18]. Landraces originating from Jordan, Syria and Iraq constitute a different genepool compared to landraces from Italy, Greece, Tunisia, Algeria and Spain [20]. Since Cyprus is located in the crossroad of this geographic area, it can be anticipated that the local genetic diversity of durum wheat landraces was shaped from all neighboring areas. In agreement with previous studies [4,6], our data support genetic proximity between landraces from Cyprus to landraces from North Africa and the Middle East. Genetic relatedness between landraces from these areas can be expected, due to the geographical proximity and the long history of trade that goes back to the early stages of plant domestication [2] and to the similar climatic conditions [10]. ‘Kyperounda’ and ‘Maurotheri’ accessions were clustered together suggesting that these are synonyms of the same landrace. The genetic differentiation of ‘Kyperounda-Maurotheri’ accessions in combination with their high heterogeneity indicates that this is a genetically distinct landrace with high intra genetic diversity.

### Intra-genetic diversity and genetic affinity of ‘Kyperounda’ accessions

Further to the observed phenotypic diversity of ‘Kyperounda’ [7,8,40], our data revealed the high genetic diversity existing within this landrace. Other studies also reported the presence of variability within landrace populations [30,33,36,38,39]. The average number of alleles per locus was comparable to the genetic diversity within the landraces ‘Barbela’ and ‘Kunduru’ [14,37]. A high number of unique genotypes was recorded, and most genotypes are present with low frequencies. Thus, sampling a few individuals per landrace will likely not be sufficient to reveal the full genetic diversity [35]. It can be speculated that natural interspecific hybridization [3,22,41] and early breeding activities based on ‘Kyperounda’ [60] might contribute to the broadening of its genetic composition and to the introgression of unique alleles. However, this hypothesis should be investigated in future studies.

For a self-fertilized (inbreeding) species, like durum wheat, fixation indices are expected to have upper limit values (+1). Lower ranges can result from the polyploidy of durum wheat, or higher than expected rates of outcrossing. Soriano et al. [6] reported fixation indices for durum wheat germplasm ranging from -0.65 to 0.99 with landraces being more heterozygous than modern varieties. Outcrossing rate of 1.3% has been reported for breed wheat landraces that it is sufficient to generate off types by contamination with foreign pollen [30]. Outcrossing of landraces accessions during regeneration should be a concern, if the conservation of genetic integrity of landraces is the goal. In the current study, fixation index values ranged from -1 (heterozygous genotypes) to +1 (homozygous genotypes) across all five loci. In general, populations 4 and 5 (landraces conserved as bulks) had threshold values (loci BARC 74, WMC104, WMS5 and WMC89). Landraces conserved as sublines on the other hand, had intermediate values due to the more even distribution of alleles across loci. It is noteworthy to stress that these populations had a mean fixation index closer to zero (0.084 for population 3) which is expected under random mating groups. Hence, conservation in sublines, when feasible, is more beneficial for retaining the full palette of alleles and a larger amount of genetic diversity.

Two Phase Model (TPM) and the Step-wise Mutation Model (SMM) revealed heterozygosity excess to Population 5, indicating that this population experiences a reduction of its effective size [47]. In this population, twenty-six plants were examined. This result suggests that, in bulk landraces accessions conserved *ex situ*, high number of seeds are required during regeneration in order to avoid bottleneck effect and the risk to lose rare alleles [30]. The latter is of particular importance since losing rare alleles can reduce the adaptive potential of landraces [47].

The highest genetic diversity among the ‘Kyperounda’ entries was observed within population 2 which was collected from a remote area located to the western part of the island, where traditional farming systems and landraces are still used. Mountainous and remote areas are considered to be the hot spots of diversity [8,33]. Adverse climatic conditions contributed to the increase of the genetic variability within the landrace ‘Haurani’ [36] and to the accumulation of high number of rare alleles in creole wheats [23]. In the present study, the more adverse climatic conditions were recorded in Athienou (Table 2ii). Population 1, which was collected from this area, showed slightly lower amounts of diversity; most likely because Athienou is located in the central plain where intensive agriculture and the use of modern varieties were applied long before the collection of the genetic material. This is further evident from the higher percentage of admixture with dwarf sublines of durum and bread wheat found in this population in field experiments. Sublines from population 1 were earlier in heading compared to the heading of the sublines of the other two populations. Early heading contributes to drought escape during grain filling in the Mediterranean Basin, and landraces originated from drier areas were found to have earlier heading [6,10]. In addition, a large number of sublines from population 1 were grouped to Cluster 1 (structure analysis), and although no clear affiliation of the populations was observed in PCoA, some genotypes from the population 1 were grouped separately at the margins of axis 1. On the other hand, sublines from populations 2 and 3 were collected from areas with similar climatic conditions and were genetically closer. These results indicate that further to the gene flow via human activities, i.e. exchange of seeds between farmers from different geographical areas and unconscious farmers’ selections [5,31–33,35], environmental conditions can influence, to some extent, the genetic and phenotypic diversity of landraces.

### Implications between genetic diversity within landrace populations and *ex situ* conservation

The genetic differentiation between accessions of individual landraces observed in the present study, which results from their dynamic nature [9,35], reinforce the concept that passport data alone is not a reliable method to eliminate duplications of landrace accessions in genebanks [33,61]. The genetic diversity in ‘Kyperounda’ accessions was higher when conserved as a group of sublines rather than as bulks. The lower genetic diversity in the accessions conserved as bulks could be attributed to the de-bulking strategy that is followed by genebanks and the expected reduction of genetic diversity with successive regeneration of bulk samples [11,30,62]. Indeed, the high number of genotypes recorded in ‘Kyperounda’ accessions, implies that plants with different genotypes might have similar phenotypes. Thus, collecting individual spike(s) representative of the phenotype during collecting or regeneration may not be adequate to retain the full palette of genes and combinations within a phenotype [11,54], and when applied the risk of losing genetic variation is high.

The results of the present study underline the ‘hidden’ genetic diversity within individual landraces conserved as sublines. Further to the molecular tools, indigenous knowledge should be employed for optimizing sampling of sublines of each particular landrace [30]. As a further step, these sublines need to be screened under their native edaphoclimatic conditions in order to reveal their phenotypic variability, particularly for agronomically important traits. Still, we acknowledge that conserving the full spectrum of the between and within genetic variability of landraces (at a regional or global level) might not be feasible due to elevated cost for conservation. Thus, in integrated regional and global conservation systems, national conservation programs should have a predominant role for the *ex situ* conservation and characterization of the within individual local landraces genetic variability.

## Supporting information

S1 Table Chromosomal location, repeat motif and annealing temperature in the set of SSR primers. (DOCX)

S2 Table Levels of polymorphism detected by SSRs in ‘Kyperounda’ accessions. (DOCX)

S3 Table Wilcoxon test for heterozygosity excess of the five ‘Kyperounda’ populations under two evolution models. (DOCX)

S1 Fig ‘Kyperounda’ sublines depicting early (A) and late (B) maturity. (TIF)

S2 Fig Variation of ‘Kyperounda’ spikes. short beak and slithly colored ear (A), short beak and intermediate colored ear (B) short beak and intesively colored ear (C), presence of hairiness on the clums and slithly colored ear (D), long beak and slithly coloured ear (E), and long beak and intermdediate colored ear (F).
(TIF)

S3 Fig Variation between and within ‘Kyperounda’ populations for ear characteristics. (TIF)
