# Supplementary material for: Genetic variability in landraces populations and the risk to lose genetic variation. The example of landrace ‘Kyperounda’ and its implications for ex situ conservation
Source: PLoS One. 2019 Oct 29;14(10):e0224255. doi: 10.1371/journal.pone.0224255 (PMC6818954; doi:10.1371/journal.pone.0224255)
Supplement: S1 Table — (DOCX) [file pone.0224255.s001.docx]

| **Primer** | **Chrom.** | **Repeat motif** | **Anneal. temper. (^o^C)** |
| --- | --- | --- | --- |
| WMS752 | 1AS/1BL | GT | 55 |
| WMS268 | 1BL | (GA)_17_TA(GA)_27_ | 57 |
| WMS312 | 2AL | (GA)_37_ | 60 |
| WMS148 | 2BL | (CA)_22_ | 60 |
| WMS619 | 2BL | (CT)_19_ | 50 |
| WMS5 | 3AL | (TC)_23_(T)_4_(GT)_12_(GA)_10_ | 55 |
| WMS155 | 3AL | (CT)_19_ | 60 |
| WMS299 | 3B | (GA)_31_(TAG)_4_ | 55 |
| WMS389 | 3BS | (CT)_14_(GT)_16_ | 60 |
| WMC161 | 4A | (GT)_25_ | 61 |
| WMC89 | 4B/4A/4D | (CA)_19_(CT)_8_ | 51 |
| WMS304 | 5AS/2A | (CT)_22_ | 57 |
| BARC74 | 5B | (GA)_13_(GATA)_7_(GA)_9_ | 60 |
| WMS540 | 5BS | (CT)_3_(CC)(CT)_16_ | 55 |
| WMS169 | 6AL | (GA)_23_ | 55 |
| WMC104 | 6BS | (GT)_31_ | 60 |
| WMC83 | 7A | (GT)_28_ | 61 |
| WMS260 | 7A | (GA)_20_ | 57 |
| WMS46 | 7B | (GA)_2_(GC)(GA)_33_ | 60 |
