# Supplementary material for: Genetic variability in landraces populations and the risk to lose genetic variation. The example of landrace ‘Kyperounda’ and its implications for ex situ conservation
Source: PLoS One. 2019 Oct 29;14(10):e0224255. doi: 10.1371/journal.pone.0224255 (PMC6818954; doi:10.1371/journal.pone.0224255)
Supplement: S2 Table — (DOCX) [file pone.0224255.s002.docx]

| **Primer** | **No of alleles** | **Nr** | **Range (bp)** | **Dj** | **Rp** | **F(Null)** | **Fis** |
| --- | --- | --- | --- | --- | --- | --- | --- |
| BARC 74 | 12 | 5 | 156-187 | 0.71 | 2.19 | 0.7406 | 0.826 |
| WMC104 | 10 | 6 | 119-182 | 0.60 | 2.32 | 0.0109 | 0.025 |
| WMS268 | 17 | 12 | 182-265 | 0.50 | 2.20 | -0.1696 | -0.433 |
| WMS5 | 10 | 5 | 95-178 | 0.58 | 1.52 | 0.9099 | 0.943 |
| WMC89 | 7 | 2 | 121-145 | 0.55 | 1.48 | -0.2453 | -0.604 |

Nr=Number of alleles with a frequency <5%, Dj: Discriminating power. Rp: Resolving power. F(Null): probability of null alleles, Fis=Inbreeding coefficient.
