# Supplementary material for: Genetic variability in landraces populations and the risk to lose genetic variation. The example of landrace ‘Kyperounda’ and its implications for ex situ conservation
Source: PLoS One. 2019 Oct 29;14(10):e0224255. doi: 10.1371/journal.pone.0224255 (PMC6818954; doi:10.1371/journal.pone.0224255)
Supplement: S3 Table — (DOCX) [file pone.0224255.s003.docx]

| Population | Mean N | Mean  k | Mean  He | Evolution models / Probability | | |
| --- | --- | --- | --- | --- | --- | --- |
|  |  |  |  | IAM | TPM | SMM |
| Pop1 | 80 | 7 | 0.665 | 0.312 | 0.953 | 1 |
| Pop2 | 104 | 8.2 | 0.738 | 0.047* | 0.687 | 1 |
| Pop3 | 106 | 7.8 | 0.649 | 0.312 | 1 | 1 |
| Pop4 | 92 | 2.6 | 0.388 | 0.078 | 0.109 | 0.5 |
| Pop5 | 52 | 2.8 | 0.478 | 0.031* | 0.031* | 0.031* |

**= p<0.05*, N= sample size, k = mean number of alleles, Infinite Allele Model (IAM), Stepwise Mutation Model (SMM), Two Phase Model (TPM)
