# Supplementary figures and images for: Genetic variability in landraces populations and the risk to lose genetic variation. The example of landrace ‘Kyperounda’ and its implications for ex situ conservation
Source: PLoS One. 2019 Oct 29;14(10):e0224255. doi: 10.1371/journal.pone.0224255 (PMC6818954; doi:10.1371/journal.pone.0224255)

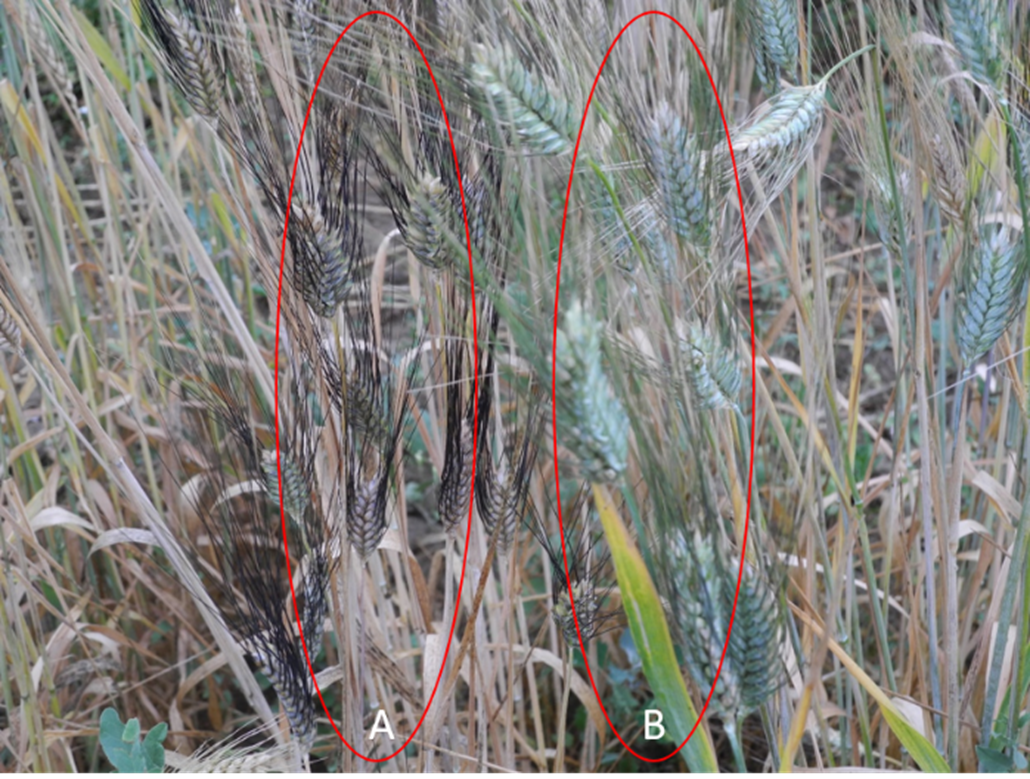

Supplement: S1 Fig — (TIF) [file pone.0224255.s004.tif]

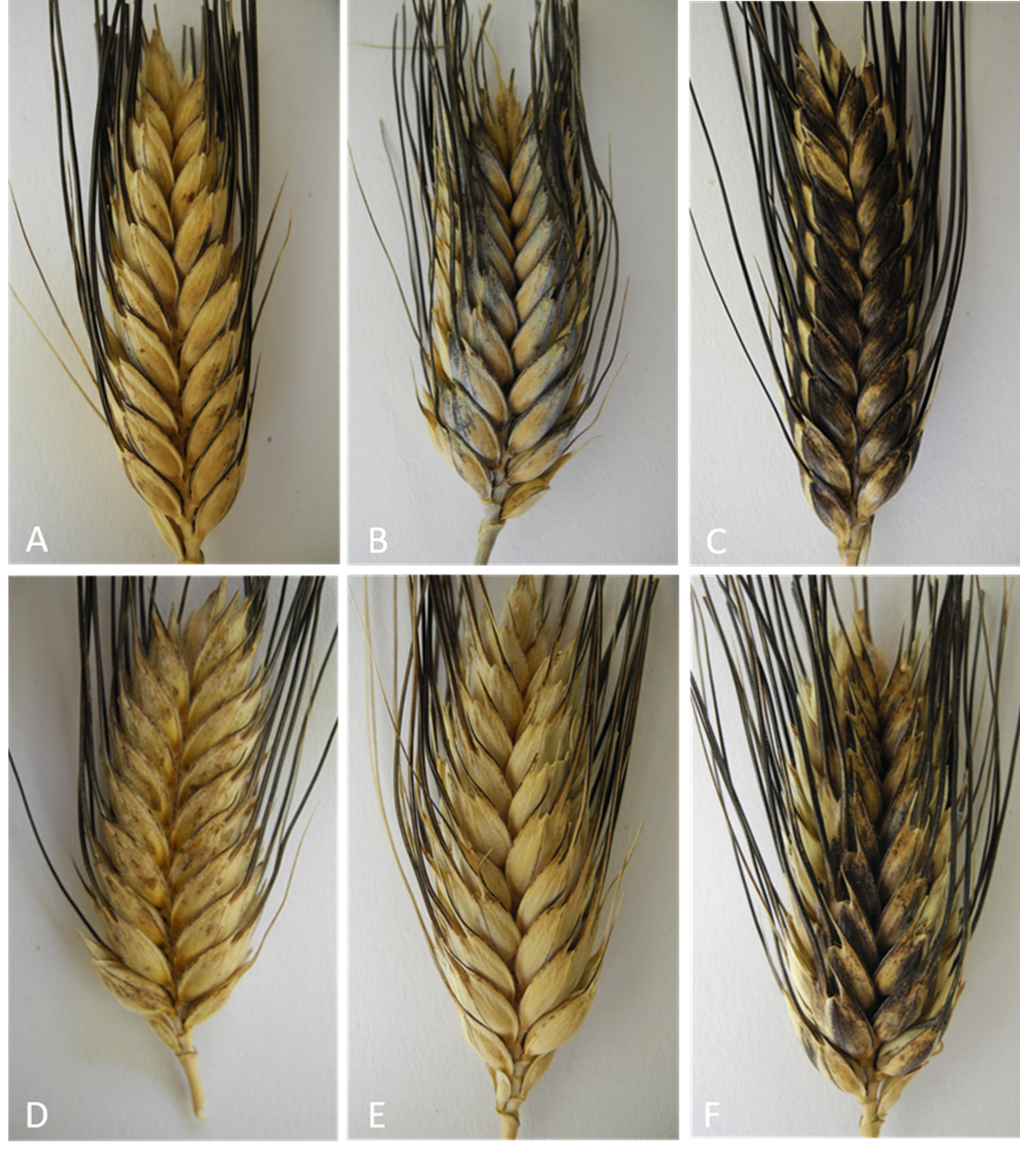

Supplement: S2 Fig — short beak and slithly colored ear (A), short beak and intermediate colored ear (B) short beak and intesively colored ear (C), presence of hairiness on the clums and slithly colored ear (D), long beak and slithly coloured ear (E), and long beak and intermdediate colored ear (F). (TIF) [file pone.0224255.s005.tif]

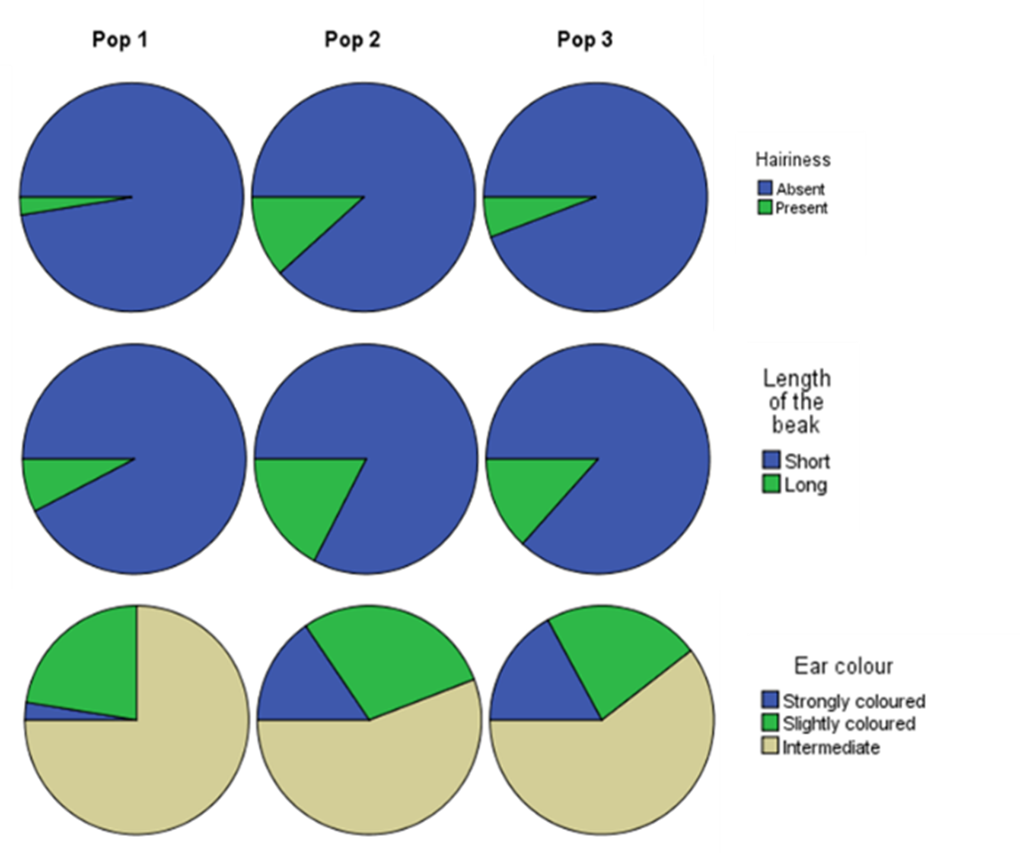

Supplement: S3 Fig — (TIF) [file pone.0224255.s006.tif]
